# The combined effect of vitamin K and calcium on bone mineral density in humans: a meta-analysis of randomized controlled trials

**DOI:** 10.1186/s13018-021-02728-4

**Published:** 2021-10-14

**Authors:** Liyou Hu, Jindou Ji, Dong Li, Jing Meng, Bo Yu

**Affiliations:** 1 Shandong University of Traditional Chinese Medicine, Jinan, 250014 China; 2 Department of Orthopaedics, Affiliated Hospital of Shandong University of Traditional Chinese Medicine, Jingshi Road 16369, Jinan, 250014 China

**Keywords:** Bone mineral density, UcOC, Vitamin K, Calcium, Systematic review

## Abstract

**Background:**

With the increasing incidence of osteoporosis, vitamin K and calcium have been linked to bone mineral density (BMD) and undercarboxylated osteocalcin (UcOC) in many studies, but the results of studies of the combined effect of vitamin K and calcium on BMD and UcOC in humans have been inconsistent. We conducted a systematic review of randomized controlled trials to assess the effect of this combination treatment on BMD and UcOC in humans.

**Methods:**

A search for articles was conducted using PubMed, Embase, and the Cochrane Library database up to March 2021 (no language restrictions). We also reviewed the reference lists of the relevant publications and reviews to locate additional publications. The standard mean difference (SMD) was used as the primary measure of effect size. Our main endpoints were lumbar BMD, femoral neck BMD, hip BMD, total femoral BMD, and UcOC from baseline to end point. We performed subgroup analysis, heterogeneity testing, and assessment of publication bias.

**Results:**

A total of 1346 patients from 10 randomized controlled trials were included in the meta-analysis. The forest plot analysis revealed that vitamin K combined with calcium was associated with a higher lumbar spine BMD compared to controls. The SMD was 0.20 [95% confidence interval (CI): 0.07 to 0.32]. Vitamin K and calcium supplementation led to a significant decrease in UcOC (SMD: − 1.71, 95% CI: − 2.45 to − 0.96). Subgroup analysis showed that vitamin K2 and vitamin K1 had SMDs of 0.30 (95% CI: 0.10 to 0.51) and SMDs of 0.14 (95% CI: − 0.02 to 0.29), and calcium dosages of ≤ 1000 mg/d or > 1000 mg/d had SMDs of 0.19 (95% CI: 0.05 to 0.32) and 0.26 (95% CI: − 0.04 to 0.55).

**Conclusion:**

The combination of vitamin K and calcium has a positive effect on lumbar BMD and decreases the level of UcOC.

*Registration*: The protocol for this meta-analysis was registered at the International Prospective Register of Systematic Reviews (CRD42021251825).

## Background

Osteoporosis is defined as a skeletal disorder characterized by compromised bone strength predisposing a person to an increased risk of fracture. Bone-related diseases, especially osteoporosis, are becoming a major public health problem as life expectancy increases [1–3]. The present studies have confirmed that many drugs can increase BMD and reduce the incidence of fractures [4, 5]. Actually, nutrition also plays a crucial role in the prevention and treatment of bone-related diseases. Taking vitamin D supplements, for example, can improve bone quality in older adults [6]. Calcium is also important. Hydroxyapatite calcium crystals deposit in the type I collagen matrix of bones, increasing bone strength. Inadequate calcium intake can lead to osteoporosis and fractures [7]. Adults can reach peak bone mass between the ages of 30 and 35, so older adults and adolescents must maintain high calcium intake [8]. In addition to vitamin D and calcium, recent studies have shown that vitamin K also has a beneficial effect on bone. Vitamin K is involved in the carboxylation of osteocalcin, thereby regulating the accumulation of bone minerals [9]. Vitamin K appears to promote the transition from osteoblasts to osteocytes while also limiting osteoclast production [10]. Vitamin K also regulates calcium use in the body, promoting calcium and bone binding [11]. There are several subtypes of vitamin K: K1, K2, K3, K4, and K5. The two main subtypes are vitamins K1 and K2, both of which are fat-soluble, allowing them to enter cells without the need for transmembrane transporters, especially in animals. The sources of the two major subtypes of vitamin K are different; vitamin K1 is found mainly in green leafy vegetables [12]. The source of vitamin K2 varies with the length of the menaquinone; MK-4 is found in animal products, and the most abundant sources of MK-7 are bacteria-fermented foods such as natto, a traditional soy dish common in Japan [13]. Recent evidence suggests that vitamin K regulates osteoblastogenesis and osteoclastogenesis through the nuclear factor κB (NF-κB) signal transduction pathway. NF-κB signaling exerts two functions: it stimulates osteoclast development and resorption and inhibits osteoblast differentiation and activity [14].

Lack of vitamin K and calcium may affect bone metabolism and eventually lead to osteoporosis and fractures. Bone material properties, the degree of mineralization, and microdamage accumulation are influenced by collagen crosslinking, specifically type I collagen fiber and crosslinking fiber, which forms the connection matrix protein and mineral crystal framework. If the arrangement of the collagen fibers changes and the mineral crystal is immature, the material properties change, which may damage bone elasticity. Vitamin K, through a nuclear steroid and xenobiotic receptor-mediated effect on the collagen content, may be an important factor that affects bone quality [15, 16]. The effect of vitamin K on bone health and remodeling also involves matrix Gla protein (MGP), which promotes bone formation by upregulating Wnt/β-catenin signaling and exerts an inhibitory effect on bone mineralization [17]. In the late stage of osteoclast differentiation, MGP is highly expressed, thus forming a negative feedback loop so that the formation of osteoclasts is strictly controlled [18]. Wu et al. [19] showed that vitamin K inhibitor osteoclast-mediated effects on bone resorption occur in a dose-dependent manner. Calcium is the most abundant inorganic salt component in the human body; 99% of it is found in bones and teeth. It is essential for the normal function of nerves and muscles and plays a role in blood clotting (as factor IV) and many enzymatic processes. Calcium is directly affected by the amount of vitamin K in the body, and when calcium metabolism is impaired, the resulting increased calcium in the arteries and decreased calcium in the bones are known as the calcium paradox [20]. Moreover, bone remodeling requires a variety of cells, including osteoblasts, osteoclasts, and osteocytes. Osteoblasts continuously synthesize the non-collagenous protein osteocalcin, which is one of the most abundant proteins in bone and is one of many vitamin K-dependent proteins, making vitamin K crucial in bone remodeling, one of its many roles in calcium metabolism [21].

Many studies have investigated the effects of vitamin K combined with calcium on bone health. However, no meta-analysis published to date has concluded that vitamin K combined with calcium has a consistent effect on bone mass. Therefore, we conducted a meta-analysis to evaluate the impact of vitamin K combined with calcium on bone BMD.

## Material and methods

### Search strategy and selection criteria

This systematic review is reported in accordance with the Preferred Reporting Items for Systematic Reviews and Meta-Analyses (PRISMA) Statement and was registered at the International Prospective Register of Systematic Reviews (CRD42021251825).

All articles that were indexed up to March 2021 and published in PubMed, Embase, and Cochrane Library were searched. The following combinations of keywords were used in the systematic search: (vitamin K OR menatetrenone OR menaquinone OR phylloquinone OR VK) AND calcium AND (postmenopausal OR BMD OR bone OR osteoporosis OR osteopenia OR osteoporotic). Articles cited in the references of identified relevant articles were used to find other publications. We included studies written in all languages.

### Study selection and data extraction

The inclusion criteria were as follows: (1) subjects aged over 18 years; (2) randomized controlled trial (RCT), conducted to compare vitamin K and calcium used in combination to a control group taking only vitamin K or calcium or a placebo; (3) the intervention entailed taking vitamin K and calcium supplements for at least six months; (4) trials providing bone-related data on lumbar BMD, total femoral BMD, femoral neck BMD, or UcOC.

The exclusion criteria were as follows: (1) participants with steroid-induced secondary osteoporosis; (2) studies without bone-related data, including total femoral BMD, femoral neck BMD, lumbar BMD, or UcOC level; (3) studies other than RCTs; (4) subjects with certain diseases or taking medications that may affect BMD.

Articles were selected and reviewed independently by two authors (DL and JM). They filtered titles and abstracts based on the relevance of the topic. After reading the abstract, the full text was screened to select the articles that were eventually included in the meta-analysis. The articles eligible for inclusion were independently selected by the two authors. When it was not clear whether an article should be included, it was discussed with a third author (JDJ) to reach a consensus.

The following information was extracted from each included study: first author’s last name; year of publication; duration of follow-up; geographical location; participant characteristics including age, sample size, and vitamin K; and calcium doses and body mass index (BMI). We also extracted the mean and standard deviation (SD) changes in lumbar BMD, femoral neck BMD, hip BMD, total femoral BMD, and UcOC from baseline to end point, or their percentage changes from baseline to end point. Differences were resolved through negotiation. If a study had more than two experiments, each experiment was analyzed as a separate study in the analysis. Two independent reviewers (DL, JM) assessed the risk of bias according to the PRISMA recommendations.

### Statistical analysis

We performed a meta-analysis by extracting data from the included studies. The results of continuous variables were expressed as the standardized mean difference (SMD) and reported with 95% confidence intervals (CIs). The Chi-square test (χ2) and I-square test (Ι2) were used to examine the heterogeneity between the studies. If *I*^2^ was > 50%, a sensitivity analysis was conducted. If *I*^2^ was still > 50% after eliminating the outlying article, a DerSimonian–Laird random-effects model was used [22]. If *I*^*2*^ was ≤ 50%, a Mantel–Haenszel fixed-effects model was used for the analysis.

In a meta-analysis, *I*^2^ is believed to be the best measure of the consistency between trials because it describes the percentage of total variation across studies due to heterogeneity rather than chance [23]. *I*^2^ values of 25%, 50%, and 75% were considered to be low, medium, and high heterogeneity, respectively [24]. We performed subgroup analyses to examine whether this might be a source of heterogeneity by examining the associations between vitamin K and calcium and study characteristics. Furthermore, we used Begg’s and Egger’s regression tests, and trim-and-fill funnel plots were used to assess publication bias [25–27]. All statistical analyses were conducted using Stata 12 (StataCorp, College Station, Texas).

## Results

### Search results

**Fig. 1:**
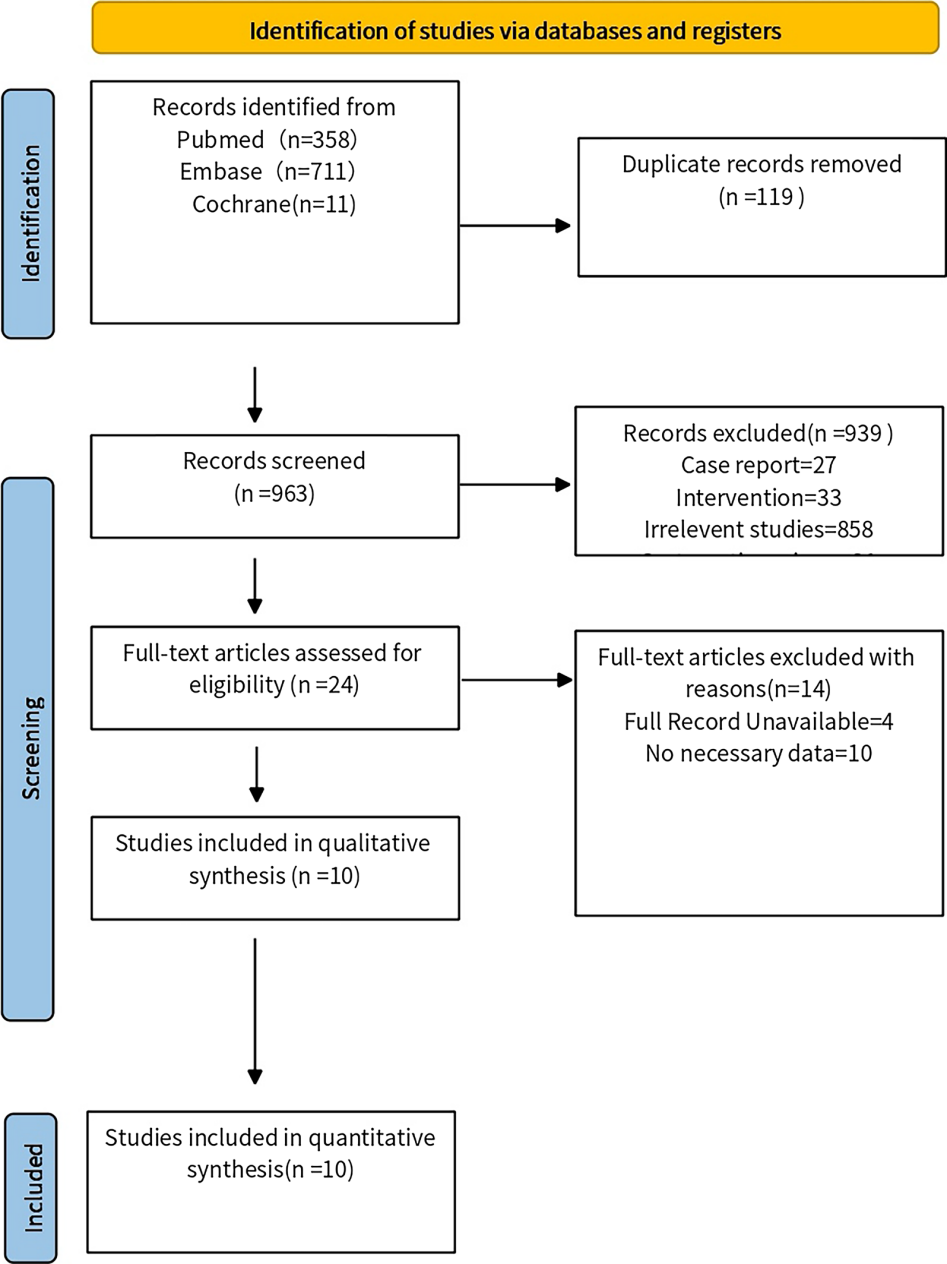
Flow chart of article selection process

Of 1,080 unique citations obtained: 357 were from PubMed, 711 from Embase, and 11 from the Cochrane Library. Finally, 10 studies were included for qualitative and quantitative syntheses [28–37], including 1346 participants. The screening steps and reasons for exclusion are shown in (Fig. 1).

### Study characteristics

The intervention duration of all studies ranged from six months to four years, and of the included studies, 7 trials were conducted with vitamin K2 and 6 with vitamin K1. Each trial reported the type and dose of vitamin K, the dose of calcium, the patients’ mean age, the BMD (based on baseline change), the mean UcOC (based on baseline percentage change), and outcomes (Table 1). The results were reported as follows: total femoral BMD (*n* = 6), femoral neck BMD (*n* = 7), lumbar vertebra BMD (*n* = 10), Hip BMD (*n* = 3), and UcOC (*n* = 10).

**Table 1:** Characteristics of eligible randomized controlled trials

Study	Year	Vitamin K/d	Ca/d	Type	Follow-up	Country	BMI(k/gm)	Age	No. (intervention/control, subjects)	Outcomes	Lumbar vertebrae BMD(mg/cm2)	Total femoral BMD(mg/cm2)	Femoral neck BMD(mg/cm2)	UcOC, based on baseline percentage change
Ronn (K2)	2020	375 μg	800 mg	K2	3 year	Denmark	NR	67.4	62/57	Femoral neck, hip	NR	NR	− 10/− 8	NR
Zhang et al. (K2)	2020	90 µg	500 mg	K2	1 year	China	23.5	65.9	81/74	Lumbar spine, femoral neck, hip	2/− 4	NR	− 3/− 3	NR
Kanellakis et al.(K2)	2012	100 μg	800 mg	K2	1 year	Greece	29.43	62	24/26	Lumbar spine, UcOC, total body	6/− 32	13/24	NR	− 23.6/28.7
Kanellakis et al. (K1)	2012	100 μg	800 mg	K1	1 year	Greece	30.06	62	26/26	Lumbar spine, UcOC, total body	16/− 32	13/24	NR	− 13.6/28.7
Je et al. (K2)	2011	45 mg	1200 mg	K2	4 year	Korea	NR	67.3	18/27	Lumbar spine, UcOC, femoral neck	10/− 8	NR	10/10	− 72.2/− 25
Moschonis et al.(K2)	2011	100 μg	800 mg	K2	1 year	Greece	29.65	62	24/39	Lumbar spine, UcOC, total body	6/− 8	13/− 1	NR	− 23.6/12.8
Moschonis et al. (K1)	2011	100 μg	800 mg	K1	1 year	Greece	30	62	26/39	Lumbar spine, UcOC, total body	16/− 8	13/− 1	NR	− 13.6/12.8
Shiraki (K2)	2009	45 mg	133 mg	K2	0.5 year	Japan	NR	68	55/49	UcOC	NR	NR	NR	− 57.1/− 25
Booth et al. (MK1)	2008	500 μg	600 mg	K1	3 year	America	NR	69	95/90	Lumbar spine, UcOC, femoral neck, total body	44/43	− 9/− 8	− 1/5	− 18.5/0.8
Booth et al.(WK1)	2008	500 μg	600 mg	K1	3 year	America	NR	68	134/133	Lumbar spine, femoral neck, total body, UcOC	9/9	− 18/− 18	− 9/− 8	− 18.7/3.1
Cheung (K1)	2008	5 mg	1500 mg	K1	4 year	Canada	26.1	58.9	33/40	Lumbar spine, UcOC, femoral neck, hip	− 3.2/− 4.9	NR	− 14.2/− 19.2	− 21.2/− 2
Bolton (K1)	2007	30 μg	1000 mg	K1	2 year	Scottish	26.15	67.8	49/56	Femoral neck, radius	NR	NR	1/0.7	NR
Purwosunu (K2)	2006	45 mg	1500 mg	K2	1 year	Indonesia	23.8	60.9	33/30	Lumbar spine, UcOC	1.74/1.4	NR	NR	− 58.3/− 9.9

### Effect of the combination of vitamin K and calcium on the lumbar spine BMD

**Fig. 2:**
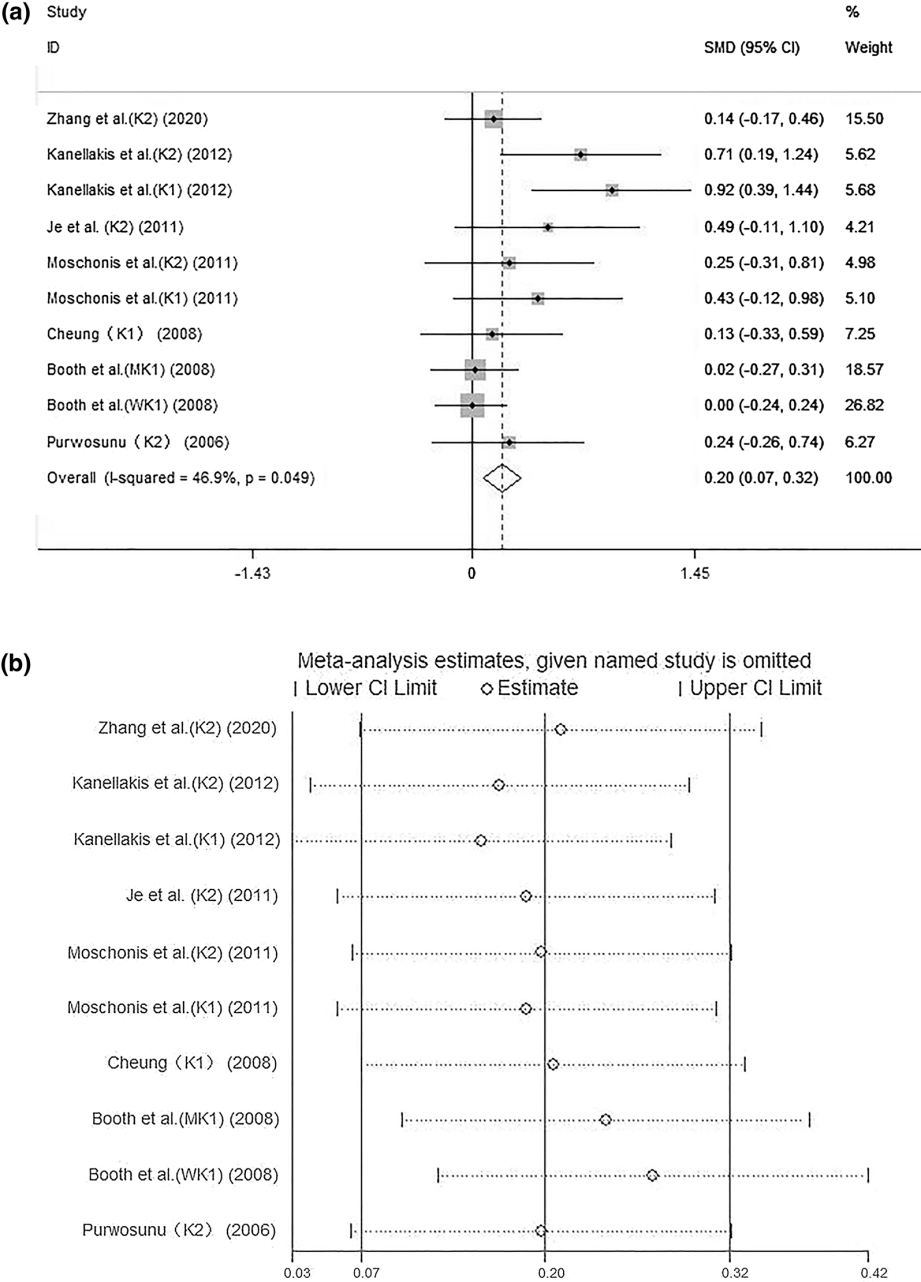
**a** Combined effect of vitamin K and calcium on lumbar bone density. **b** Sensitivity analysis of vitamin K and calcium on lumbar bone density

The forest plot (Fig. 2a) showed that the combination of vitamin K and calcium was associated with a higher lumbar spine BMD (SMD: 0.20, 95% CI: 0.07–0.32). There was low heterogeneity across studies (*I*^2^: 46.9%, *P* = 0.049). The sensitivity analysis of vitamin K and calcium on lumbar bone density is illustrated in (Fig. 2b).

### Effect of the combination of vitamin K and calcium on the total femoral BMD

**Fig. 3:**
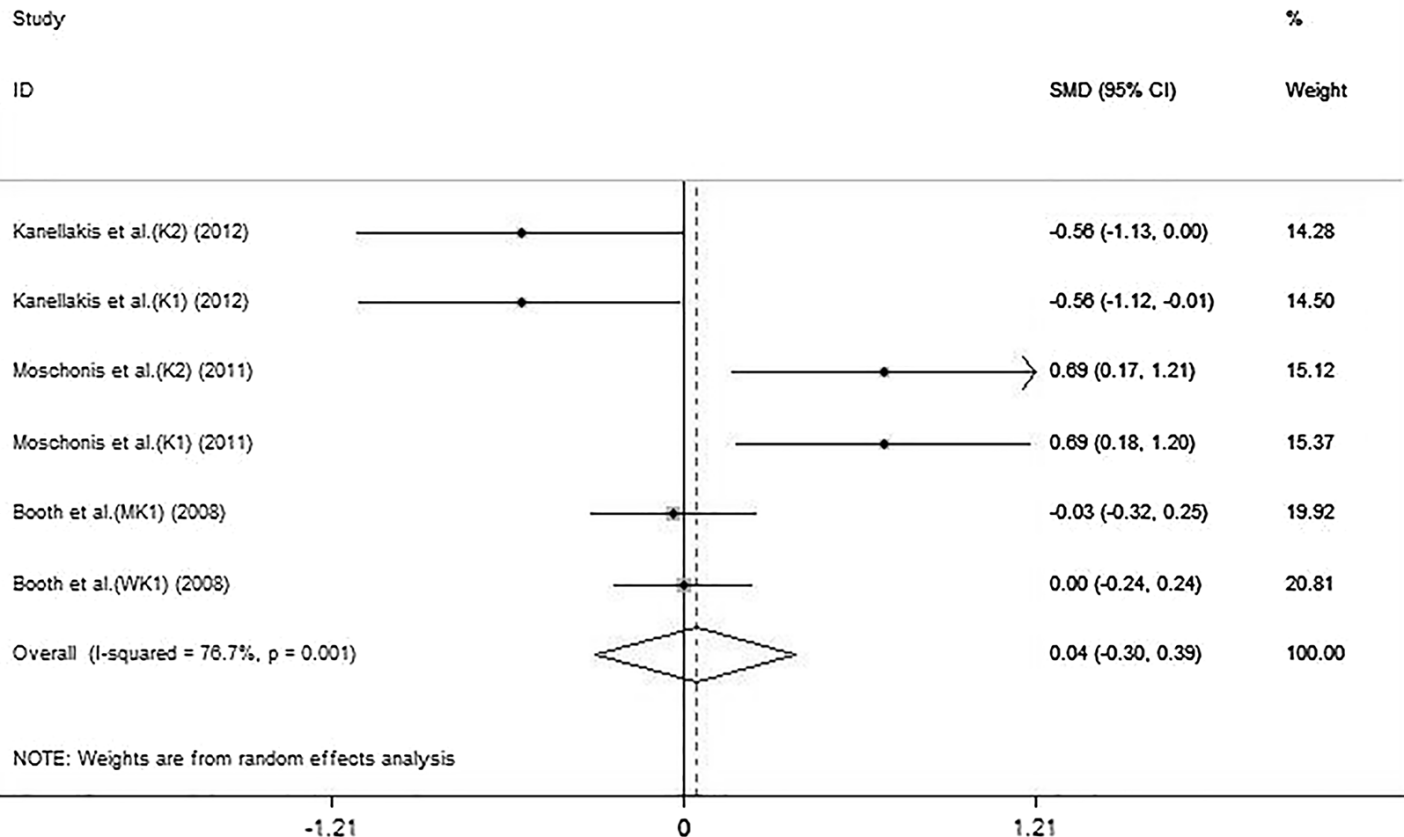
Effect of the combination of vitamin K and calcium on total femoral BMD

The forest plot (Fig. 3) showed that the combination of vitamin K and calcium did not positively affect the total femoral BMD (SMD: 0.04, 95% CI: − 0.30 to 0.39). The 6 sets of results showed a statistically heterogeneity across studies (*I*^2^: 76.7%, *P* = 0.001).

### Effect of the combination of vitamin K and calcium on the femoral neck BMD

**Fig. 4:**
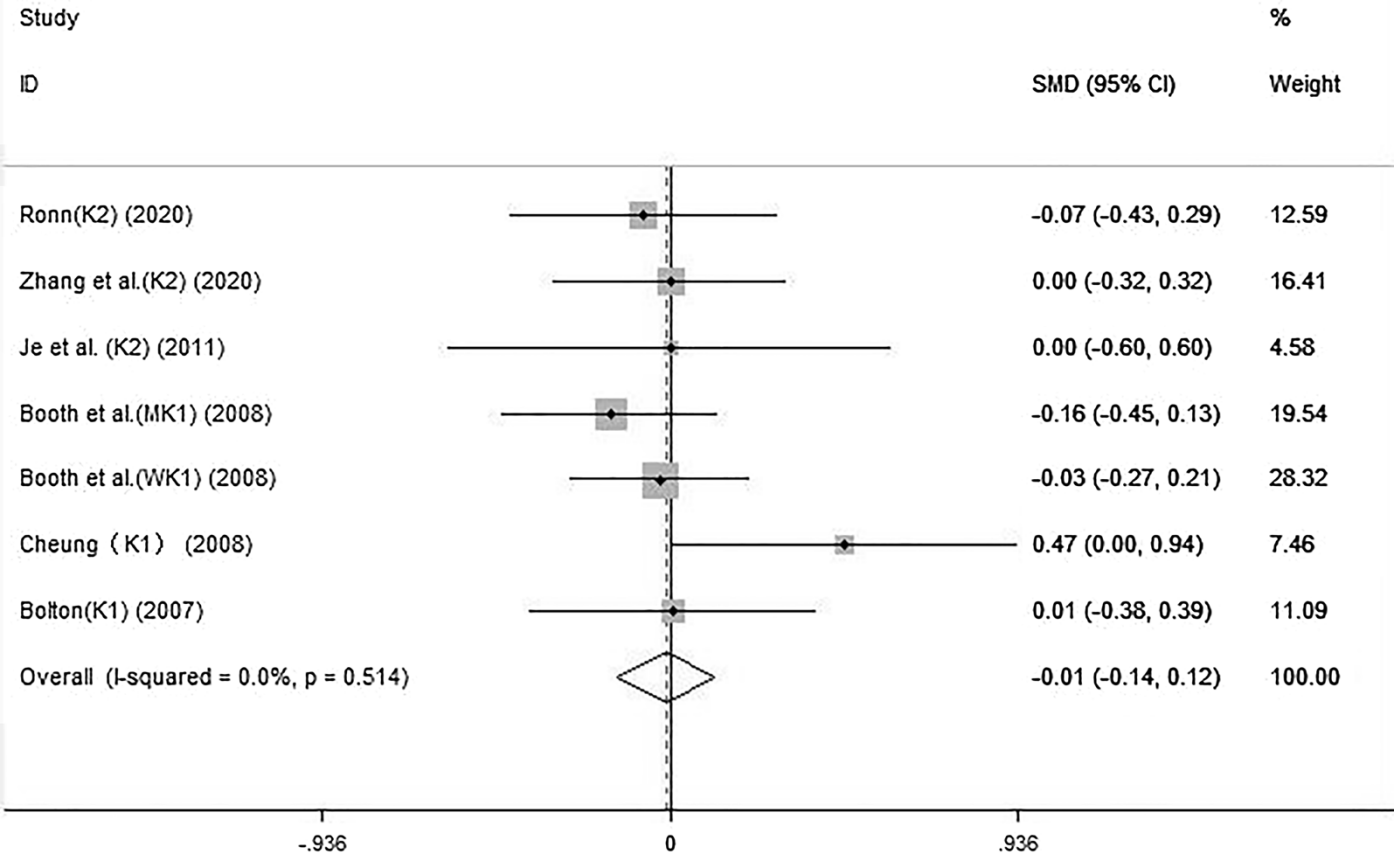
The effect of the combination of vitamin K and calcium on femoral neck BMD

The forest plot (Fig. 4) showed that the combination of vitamin K and calcium was not associated with a change in the femoral neck BMD (SMD: − 0.01, 95% CI: − 0.14 to 0.12). There was low heterogeneity across studies (*I*^2^: 0%, *P* = 0.514).

#### Effect of the combination of vitamin K and calcium on UcOC

**Fig. 5:**
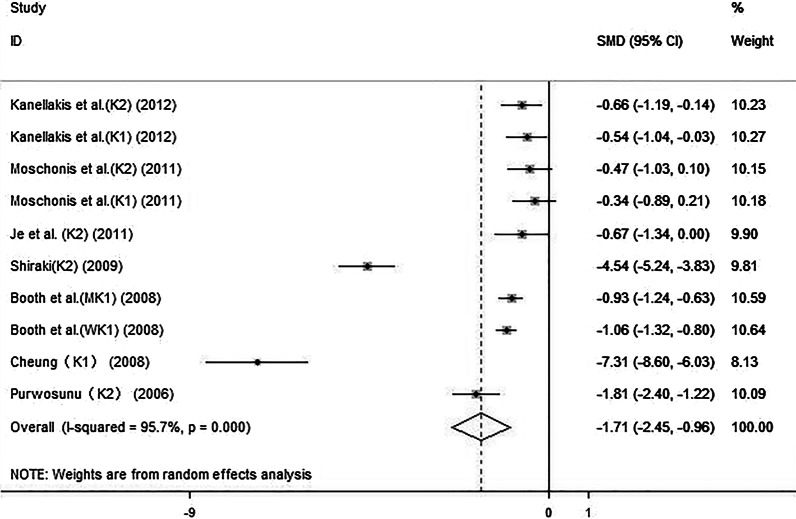
Effect of the combination of vitamin K and calcium on UcOC

The forest plot (Fig. 5) showed that vitamin K and calcium supplementation led to a significant decrease in the UcOC (SMD: − 1.71, 95% CI: − 2.45 to − 0.96). The 10 sets of results showed a statistically heterogeneity across studies (*I*^2^: 95.7%, *P* < 0.01). After the sensitivity analysis, excluding the studies by Shiraki et al. [32] and Cheung et al. [34], the SMD was − 0.82 (95% CI: − 1.10 to − 0.55). There was middle heterogeneity across studies (*I*^2^: 65.4%, *P* < 0.01).

#### Subgroup analysis

A subgroup analysis of the combination of vitamin K and calcium on lumbar spine BMD was conducted: the results are shown in (Table 2). When vitamin K supplementation was ≤ 100 µg/d (*n* = 5) or > 100 µg/d(*n* = 5), the combined effect size was SMD: 0.40 (95% CI: 0.20 to 0.61, I2: 49.9%) and SMD: 0.08 (95% CI: − 0.08 to 0.23, I2: 0%), respectively. When the type of vitamin was K2 (*n* = 5) or K1 (*n* = 5), the combined effect size was 0.30 (95% CI: 0.10 to 0.51, I2: 0%) and 0.14 (95% CI: − 0.02 to 0.29, I2: 65.4%), respectively. When the supplementation of calcium dosage was ≤ 1000 mg/d (*n* = 7) or > 1000 mg/d (*n* = 3), the SMDs were 0.19 (95% CI: 0.05 to 0.32, I2: 62.3%) and 0.26 (95% CI: − 0.04 to 0.55, I2: 0%), respectively. When the follow-up time was ≤ 1 year or > 1 year, the SMDs were 0.38 (95% CI: 0.19 to 0.57, I2: 40%) and 0.06 (95% CI: − 0.10 to 0.22, I2: 0%), respectively.

**Table 2:** Subgroup analysis to investigate the effect of the type of vitamin K on the effect size of vitamin K combined with calcium on 431 lumbar spines BMD

Subgroup	Studies	SMD (95% CI)	*I*^2^ (%)	*P*
Vitamin K type				
K1	5	0.14 (− 0.02 to 0.29)	65.4	0.021
K2	5	0.30 (0.10 to 0.51)	0	0.429
Vitamin K dose				
≤ 100 μg	5	0.40 (0.20 to 0.61)	49.9	0.092
> 100 μg	5	0.08 (− 0.08 to 0.23)	0	0.584
Calcium dose				
≤ 1000 mg	7	0.19 (0.05 to 0.32)	62.3	0.014
> 1000 mg	3	0.26 (− 0.04 to 0.55)	0	0.644
Follow-up time				
≤ 1 year	6	0.38 (0.19 to 0.57)	40	0.139
> 1 year	4	0.06 (− 0.10 to 0.22)	0	0.496
Gender				
Female	8	0.26 (0.11 to 0.41)	52.5	0.040
Both	2	0.08 (− 0.14 to 0.29)	0	0.573

#### Publication bias

**Fig. 6:**
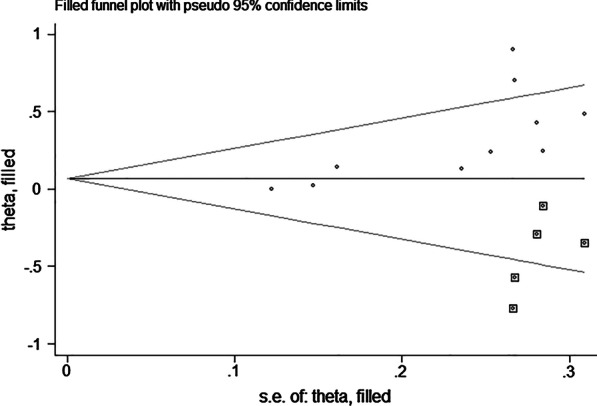
Trim-and-fill funnel plot of a meta-analysis of changes in lumbar bone density with vitamin K and calcium

Begg’s test (*P* = 0.009, *Z* = 2.59), Egger’s test (*P* = 0.049), and the asymmetry of the funnel graph suggested that there may be publication bias in assessing the combined effect of calcium and vitamin K on BMD. In order to further assess the publication bias, we adopted a trim filling method. The results revealed that there was publication bias toward underestimating the effect of vitamin K and calcium on BMD. After applying trim and fill, the results were not statistically significant (estimate: 0.067, 95% CI: − 0.044 to 0.178) (Fig. 6).

**Fig. 7:**
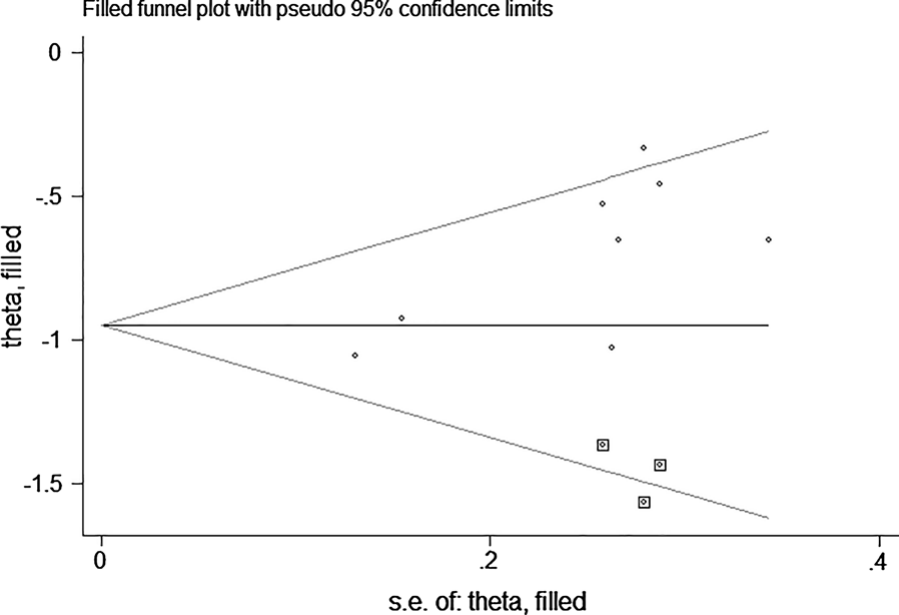
Trim-and-fill funnel plots of meta-analysis of undercarboxylated osteocalcin percentage changes with vitamin K combined with calcium

Begg’s test (*P* = 0.138, *Z* = 1.48) and Egger’s test (*P* < 0.001) for the effect of vitamin K combined with calcium on the UcOC level did not suggest the presence of publication bias. In order to further evaluate the presence of publication bias, we adopted the method of pruning and filling. The results did not change after correcting publication bias (estimate: − 0.947, 95% CI: − 1.211 to − 0.687) (Fig. 7).

## Discussion

This meta-analysis based on 1346 participants found that vitamin K and calcium used in combination were associated with a higher lumbar spine BMD and might reduce the UcOC level. To our knowledge, this is the first meta-analysis of the combined effect of vitamin K and calcium on BMD, which may provide a potential therapeutic strategy to improve lumbar BMD. To further explore inter-study heterogeneity, we performed a subgroup analysis based on vitamin K dose, type of vitamin K, calcium dose, and follow-up duration.

Some studies have found that low plasma vitamin K concentrations are associated with a higher fracture risk [38–40], but a few studies have found that vitamin K did not seem to have a beneficial effect [41]. In the past few years, most research has focused on the effect of vitamin K on the remodeling of bones, which yielded limited clinical application, though it improved the understanding of this vitamin [42]. A meta-analysis revealed that the modest overall effect of vitamin K on BMD may be biased and should be interpreted with caution [43]. Globally, vitamin K is not recommended for postmenopausal bone loss treatment. However, in Japan, vitamin K supplements have been widely recommended for the prevention of osteoporosis and subsequent fractures [44].

Many studies only focused on vitamin K alone and may have ignored the role of calcium. There have been previous meta-analyses conducted on the effects of vitamin K on BMD. Mott et al. [45] conducted a meta-analysis of 22 RCTs evaluating the effect of oral vitamin K on adult BMD and found that vitamin K was not associated with BMD changes. Kuang et al. [46] conducted a meta-analysis of eight RCTs to assess the influence of combined vitamin K and vitamin D on adult bone density, and it was found that combined use of vitamin K and vitamin D can increase bone density. The reason for the difference may be the difference in the calcium included in the two articles. In the two articles included in this study, many patients ingested different doses of calcium. Therefore, we conducted a meta-analysis to evaluate the impact of vitamin K combined with calcium on BMD.

There was high heterogeneity in the 10 UcOC studies, and after sensitivity analysis, studies by Shiraki and Itabashi [32] and Cheung et al. [34] were excluded, the remaining studies had a moderate heterogeneity, and the SMD declined. The studies by Shiraki and Itabashi and Cheung et al. were the sources of heterogeneity. The former may be due to regional variation in diet and lifestyle, and the latter may be due to taking a large amount of vitamin K1. In the study by Cheung et al., participants received up to 5 mg/d of vitamin K1, while the rest of the studies used doses of ≤ 500 µg daily. The difference in dosages may be the source of heterogeneity.

To explore inter-study heterogeneity of the combination of vitamin K and calcium on lumbar spine BMD, we performed a subgroup analysis based on the vitamin K dose, vitamin K type, calcium dose, gender, and follow-up time. When the participant was female, calcium dosage was ≤ 1000 mg/d, and the vitamin K dosage was ≤ 100 μg, the combination with calcium was associated with a higher lumbar spine BMD, but the heterogeneity was increased; this does not mean these factors are the source of heterogeneity. In the subgroup analysis of the type of vitamin K, the heterogeneity was increased. When the follow-up time was ≤ 1 year, the heterogeneity was decreased, suggesting that the follow-up time might be a source of heterogeneity.

Our meta-analysis showed that the combination of vitamin K and calcium was associated with a higher lumbar spine BMD and reduced UcOC levels. However, our study also had some limitations. First, the sample sizes of these studies varied greatly, which may be one reason for the high heterogeneity. However, patients included in the study may come from the same location or hospital, further reducing heterogeneity. To explore the sources of heterogeneity, we performed a subgroup analysis and found that follow-up time may be the source of heterogeneity. Unfortunately, due to the diversity of studies, we cannot eliminate heterogeneity completely. Furthermore, few studies have focused on the relationship between vitamin K combined with calcium and BMD. Hence, the data collected are minimal, which leads to our conclusion that evidence for the relationship between the combination of vitamin K and calcium on BMD is not sufficient. Finally, the studies included may have been influenced by demographic characteristics, limitations, and other factors. For these reasons, we recommend that our conclusions are interpreted conservatively.

## Conclusion

Our study indicates that the combination of vitamin K and calcium has a positive effect on lumbar BMD and decreases the level of UcOC. Vitamin K combined with calcium may be a potential therapeutic strategy to improve lumbar BMD.

## Data Availability

The datasets used and/or analyzed during the current study are available from the corresponding author on reasonable request. The protocol for this meta-analysis was registered on PROSPERO (CRD42021251825) and is available at: https://www.crd.york.ac.uk/PROSPERO.

## Abbreviations

BMD: Bone mineral density
Ca: Calcium
CI: Confidence interval
MGP: Matrix Gla protein
NF-κB: Nuclear factor κB
PRISMA: Preferred Reporting Items for Systematic Reviews and Meta-Analyses
RCT: Randomized controlled trial
SMD: Standardized mean difference
UcOC: Undercarboxylated osteocalcin
